# miR-132/212 Knockout Mice Reveal Roles for These miRNAs in Regulating Cortical Synaptic Transmission and Plasticity

**DOI:** 10.1371/journal.pone.0062509

**Published:** 2013-04-26

**Authors:** Judit Remenyi, Mirjam W. M. van den Bosch, Oleg Palygin, Rajen B. Mistry, Colin McKenzie, Andrew Macdonald, Gyorgy Hutvagner, J. Simon C. Arthur, Bruno G. Frenguelli, Yuriy Pankratov

**Affiliations:** 1 Wellcome Trust Centre for Gene Regulation, College of Life Sciences, University of Dundee, Dundee, United Kingdom; 2 MRC Protein Phosphorylation Unit, College of Life Sciences, University of Dundee, Dundee, United Kingdom; 3 School of Life Sciences, University of Warwick, Coventry, United Kingdom; 4 Institute of Molecular and Cellular Biology, Faculty of Biological Sciences, University of Leeds, Leeds, United Kingdom; 5 Divsion of Cell Signalling and Immunology, College of Life Sciences, University of Dundee, Dundee, United Kingdom; The John Curtin School of Medical Research, Australia

## Abstract

miR-132 and miR-212 are two closely related miRNAs encoded in the same intron of a small non-coding gene, which have been suggested to play roles in both immune and neuronal function. We describe here the generation and initial characterisation of a miR-132/212 double knockout mouse. These mice were viable and fertile with no overt adverse phenotype. Analysis of innate immune responses, including TLR-induced cytokine production and IFNβ induction in response to viral infection of primary fibroblasts did not reveal any phenotype in the knockouts. In contrast, the loss of miR-132 and miR-212, while not overtly affecting neuronal morphology, did affect synaptic function. In both hippocampal and neocortical slices miR-132/212 knockout reduced basal synaptic transmission, without affecting paired-pulse facilitation. Hippocampal long-term potentiation (LTP) induced by tetanic stimulation was not affected by miR-132/212 deletion, whilst theta burst LTP was enhanced. In contrast, neocortical theta burst-induced LTP was inhibited by loss of miR-132/212. Together these results indicate that miR-132 and/or miR-212 play a significant role in synaptic function, possibly by regulating the number of postsynaptic AMPA receptors under basal conditions and during activity-dependent synaptic plasticity.

## Introduction

miRNAs are small 20 to 22 base RNA species that are involved in the post transcriptional regulation of protein expression. miRNAs have been implicated in a wide range of processes ranging from cell proliferation and differentiation to the modulation of specific neuronal and immune function. In mammalian cells miRNAs typically interact with their target mRNAs via a 7 to 8 base seed sequence that is complementary to the target mRNA. This allows the miRNA to repress the expression of its targets either by inhibiting translation or promoting RNA degradation (reviewed in [1], [2]).

miR-132 and miR-212 are two related miRNAs that are encoded from the same intron of a small non-coding gene that is located on chromosome 11 in mice and chromosome 17 in humans. In cells, the transcription of the primary transcript for miR-132 and miR-212 can be induced by a variety of signals, including BDNF stimulation and synaptic activity in neurons, PMA and anisomycin in fibroblasts and LPS in THP-1 cells [3], [4], [5], [6], [7], [8]. The transcription of pri-miR-132/212 is regulated by CREB, and is reduced by inhibitors or genetic manipulations that block CREB phosphorylation [3], [4]. Processing of the pri-miR-132/212 transcript has been shown to give rise to 4 miRNA species; miR-132, miR-212 as well as the star sequences for both miR-132 and miR-212 [3]. Interestingly while miR-132 and miR-212 have similar seed sequences, suggesting they could have some targets in common, the seed sequences of miR-132* and miR-212* are different suggesting they would have distinct targets *in vivo*. While it is clear that all 4 miRNA sequences are expressed from this locus, the expression level for miR-132 is much higher that for the other three miRNAs [3], [9]. This has led to the suggestion that miR-132 is the only functional miRNA expressed from this locus in neurons [9], however this may not reflect the situation in all cell types.

Several targets have been proposed for miR-132 or miR-212, including MeCP2, p250GAP, p120GasGAP, p300, SirT1, Foxp2 and MMP9 [4], [10], [11], [12], [13], [14], [15]. Overexpression of miRNA mimetics or inhibitory oligos for miR-132 has suggested roles for miR-132 in dendritic branching and spine formation in neurons [4], [16], [17], [18], angiogenesis during tumour growth [10] and in the regulation of immunity and viral replication [11], [19], [20]. In addition, deletion of miR-132 and miR-212 in mice has shown a role for these miRNAs in mammary gland development [12] and cardiovascular function [21]. *In vivo* miR-132 and miR-212 have been linked to several processes in the brain including circadian rhythms, cocaine addiction and ocular dominance [22], [23], [24], [25], [26].

Much of the initial work on miR-132 and miR-212 function has relied on the use of the overexpression of miRNA mimetics or inhibitors. While these provide a powerful way to start to dissect the function of a specific miRNA, it is possible they may give rise to non-physiological off-target effects. These could occur for several reasons, such as the presence of the miRNA in a situation when it would not normally be expressed, or the overloading of Ago proteins with the miRNA mimetic thus affecting the pool of Ago available to endogenous miRNAs. Thus, the use of genetic manipulation in mice provides a powerful method to complement earlier expression based studies. Therefore, to further examine miR-132/212 function we generated a knockout mouse lacking these two miRNAs.

## Results

### Generation of miR-132/212 Knockout Mice

**Figure 1:**
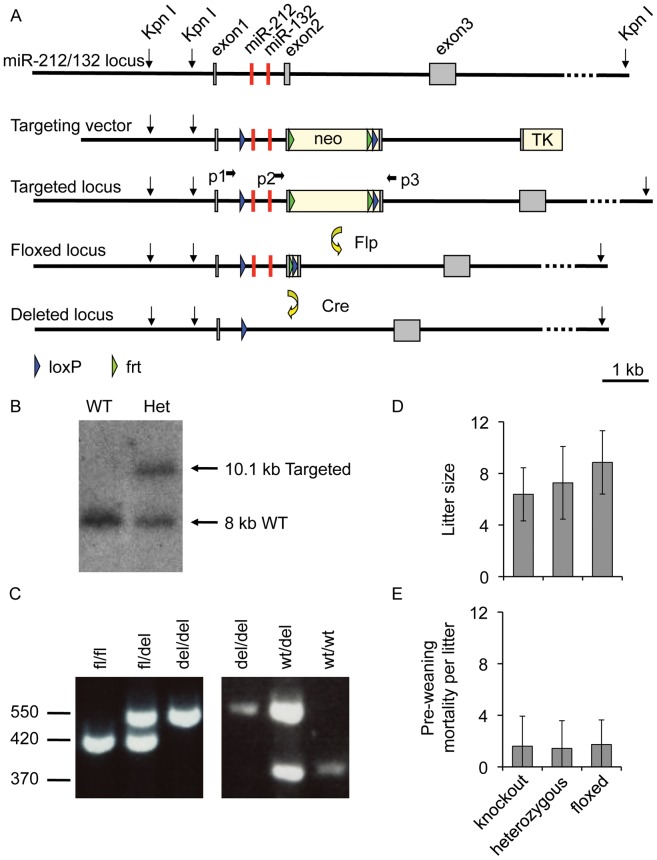
Generation of miR-132/212 knockout mice. miR-132/212 knockout mice were generated by insertion of LoxP sites in the 1^st^ intron and exon2 of the small non coding RNA gene that contains miR-132 and miR-212 (A). Targeted C57BL/6 ES cells were generated as described in the Methods section, and identified by Southern analysis of Kpn I digests using a probe 3′ to the sequence used in the targeting vector (B). Correctly targeted ES cells were used to generate a conditional miR-132/212 allele in mice using standard techniques. Mice were crossed to Flp transgenic mice to excise Neomycin resistance cassette and then Cre expressing mice to delete miR-132 and miR-212. Routine genotyping was carried out using 3 primers (p1, p2 and p3) which resulted in bands of 373 bp for the wild-type allele, 420 for the floxed allele and 550 for the deleted allele (C). Litter sizes (D) and pre weaning mortality (E) for matings of male and female knockout (n = 50 for litter size, n = 39 for pre weaning mortality), floxed (n = 20 or 17) or heterozygous (n = 53 or 41) mice are shown. Litters where pups were used for neuronal cultures were excluded from the pre weaning mortality values. Error bars represent standard deviation.

In mice, miR-132 and miR-212 are encoded in the 1^st^ intron of a small non-coding gene on chromosome 11. Conditional knockout mice for miR-132 and miR-212 were generated by insertion of loxP sites in the 5′ region of the intron encoding miR-132 and miR-212 and in exon2 using the targeting strategy shown in Fig. 1A. ES cell targeting was carried out in ES cells derived from C57Bl/6N mice using standard protocols, and correctly targeted ES cell clones were identified by Southern blots using a probe external to the targeting vector (Fig. 1B). A positive ES cell clone was used to generate germline transmitting chimeric mice, which were crossed to Flp transgenic mice to excise the neomycin cassette. Mice bearing this floxed allele were then further crossed to constitutive Cre expressing mice, resulting in heterozygous knockout alleles for the miR-132/212 locus. The genotype of mice was confirmed by PCR genotyping of ear biopsies (Fig. 1C). Crossing of heterozygous miR-132/212 knockout mice showed that the homozygous miR-132/212 knockout mice were viable and obtained at close to the expected Mendelian frequency (23.3%, n = 172). miR-132/212 knockout mice were fertile, and gave similar litter sizes and survival of pups to weaning as either heterozygous crosses or homozygous matings for the floxed allele (Fig. 1D and E). As the total miR-132/212 knockout was viable, these mice were used for subsequent studies.

**Figure 2:**
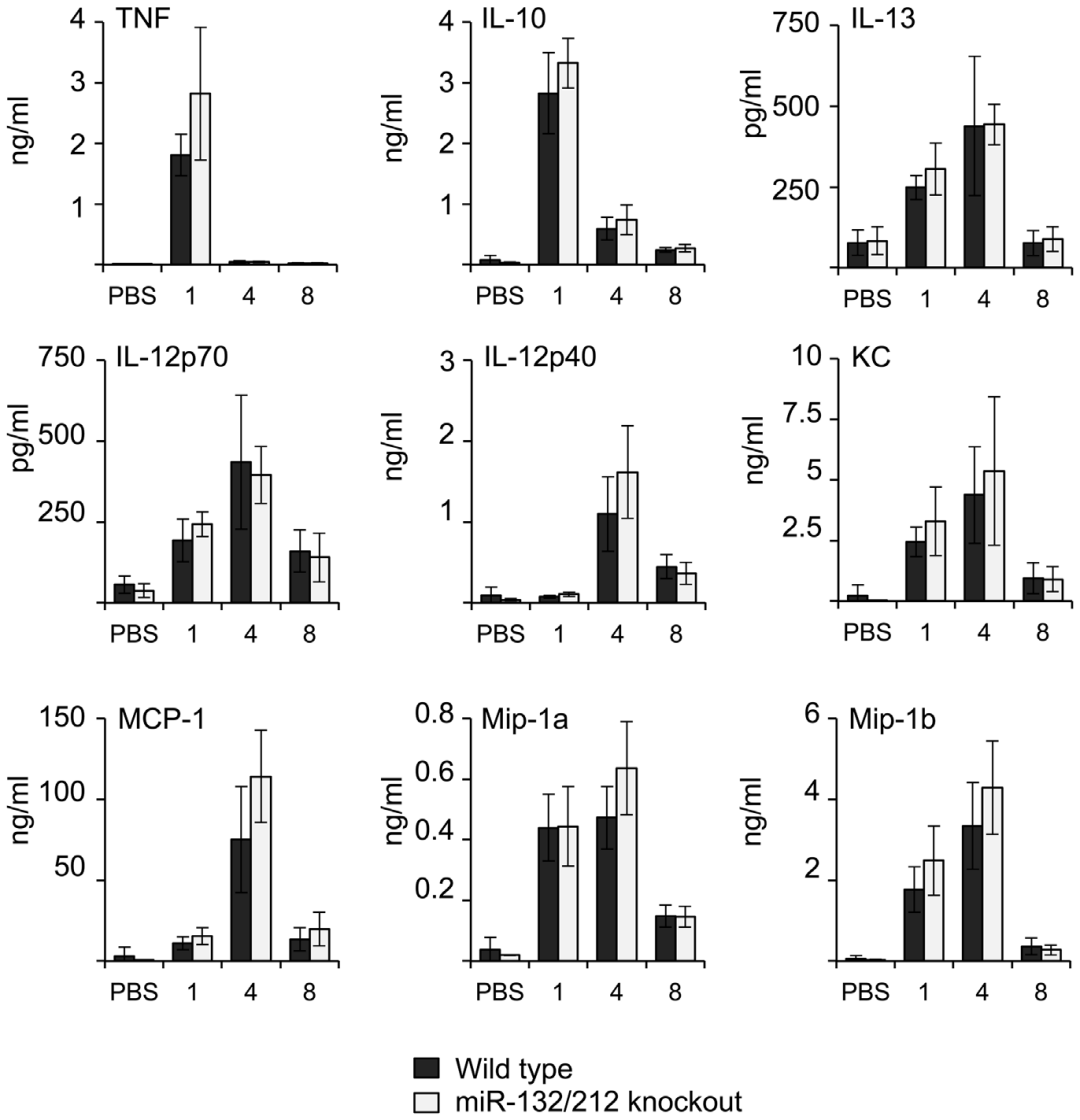
Expression or miR-132 and miR-212 in the CNS. Total RNA was isolated from the cortex (A) or cerebellum (B) of wild-type (+/+), miR-132/212 floxed (fl/fl), miR-132/212 knockout (−/−) mice. The levels of mature miR-132 and miR-212 were determined by qPCR. For the cortex samples, the levels of the mRNAs for p250Gap, MeCP2 and p300 were also determined (C). Error bars represent the standard deviation of 5 mice. A p value (students t-test) of <0.05 is indicated by * and <0.01 by **.

To confirm that the knockout did not express either miR-132 or miR-212, total RNA was isolated from cortex or cerebellum of the mice, and analysed for the expression of mature miR-132 and miR-212 by qPCR (Fig. 2A, B). Both wild-type and mice homozygous for the floxed allele had comparable levels of both miR-132 and miR-212, indicating that the insertion of the loxP sites did not affect the expression of the two miRNAs. No miR-132 or miR-212 expression could be detected in the miR-132/212 knockout brain tissue. Previously we have shown that in cortical neuronal cultures miR-132 expression is regulated by an MSK-CREB dependent pathway in response to neurotrophins [3], while others have shown a strong CREB dependence for miR-132 is cell culture systems [4]. Interestingly expression of both miR-132 and miR-212 could be detected in the cortex and cerebellum of either MSK1/2 knockout or CREB Ser133Ala knockin mice, although there was a trend for reduced levels in the cortex. This indicates that other mechanisms in addition to the MSK-CREB dependent pathway promote miR-132 and miR-212 expression during development of the CNS. Analysis of the mRNA levels in the cortex for the potential miR-132 targets p250GAP, MeCP2 and p300 did not demonstrate any significant difference between the knockout and floxed mice (Fig. 2C).

### Normal Innate Immune Responses in miR-132/212 Knockout Mice

**Figure 3:**
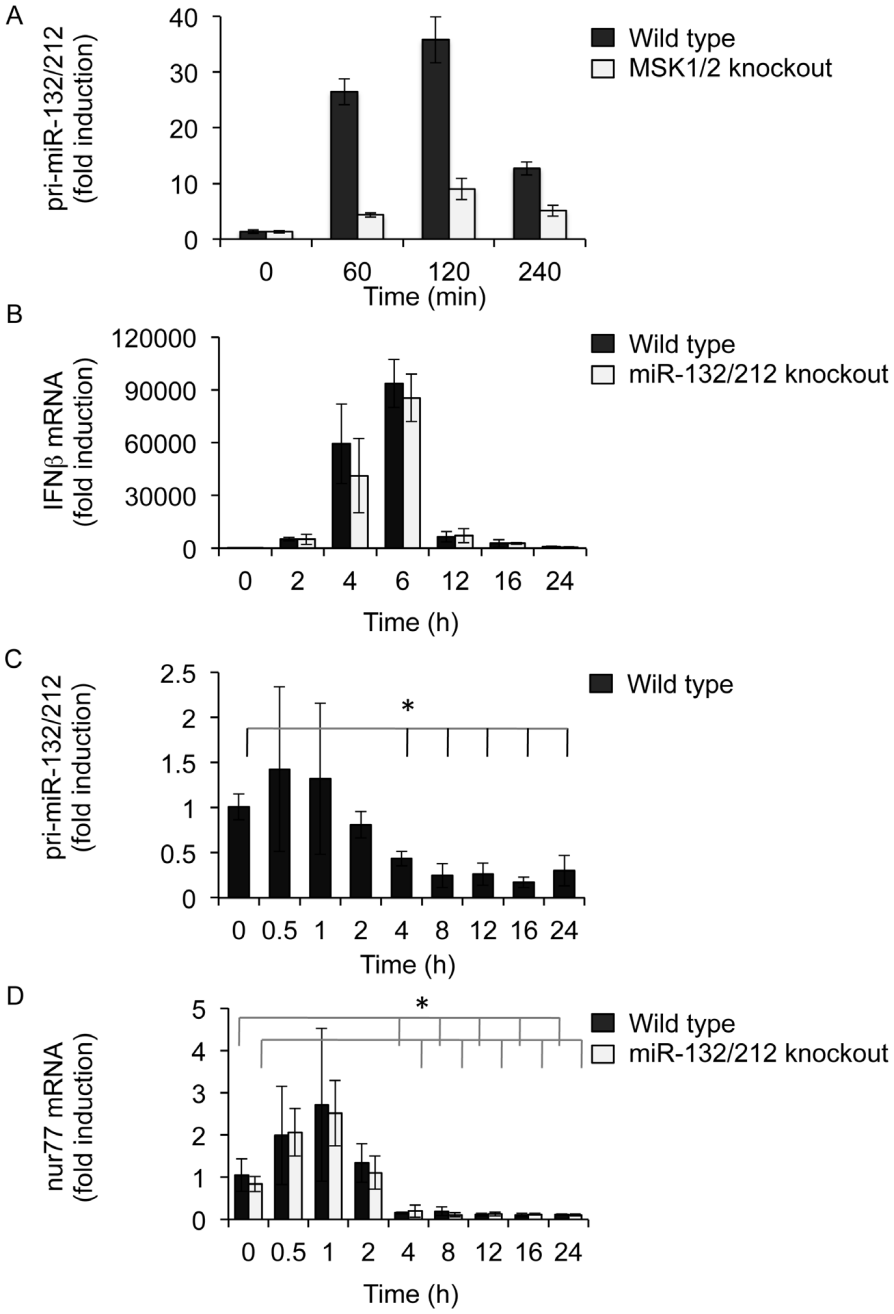
miR-132 and miR-212 are not critical for IFNβ induction in MEFs infected by Sendai virus. MEFs were isolated from wild-type or MSK1/2 double knockout mice and stimulated for the indicated times with 400 ng/ml PMA. Total RNA was isolated and pri-miR-132/212 levels determined by qPCR as described in the methods (A). MEFs from wild-type or miR-132/212 knockout mice were infected with Sendai virus for the indicated times. Total RNA was isolated and the levels of IFNβ (B), pri-miR-132/212 (C) and nur77 (D) determined by qPCR. In each case fold stimulation was calculated relative to the unstimulated wild-type samples, and error bars represent the standard deviation of 4 independent cultures from 4 mice per genotype. For (C) and (D) *indicates a p value of <0.05 relative to the unstimulated control sample.

Previous studies have suggested roles for miR-132 in the regulation of innate immunity. In response to viral infection, miR-132 has been suggested to control CREB-dependent signalling by targeting the CREB co-activator p300 [13]. In addition, miR-132 has also been suggested to regulate sirT1, a deacetylase that regulates NFκB, a transcription factor central to the production of cytokines in response to TLR agonists [11]. Initially we examined the role of miR-132 and miR-212 in mouse embryonic fibroblasts. Pri-miR-132/212 could be induced in MEFs in response to PMA, an activator of the ERK1/2 MAPK pathway (Fig. 3A). In response to PMA, ERK1/2 activate MSKs which in turn phosphorylate the transcription factor CREB [27]. Consistent with previous studies in primary neurons [28], pri-miR-132/212 induction was reduced, but not abolished, in MSK1/2 double knockout MEFs (Fig. 3A).

We next tested the effect of viral infection using Sendai virus. Infection of MEFs with Sendai virus induced an anti-viral response in wild-type cells, as demonstrated by the induction of IFNβ transcription (Fig. 3B). Infection with Sendai virus did not induce a strong activation of MAPKs or CREB in MEFs (data not shown). Consistent with this, little induction of pri-miR-132/212 was seen and, by 4 h post infection, the levels pri-miR-132/212 were actually repressed relative to uninfected controls (Fig. 3C). In line with this, at 1 h post infection with Sendai virus there was only a small increase in the mRNA levels of another CREB-dependent gene, nur77. Similar to pri-miR-132/212, this was followed by a repression of nur77 mRNA levels at later time points after infection (Fig. 3D). The effects of Sendai virus infection on IFNβ or nur77 mRNA was unaffected by the knockout of miR-132 and miR-212 (Fig. 3B and D).

**Figure 4:**
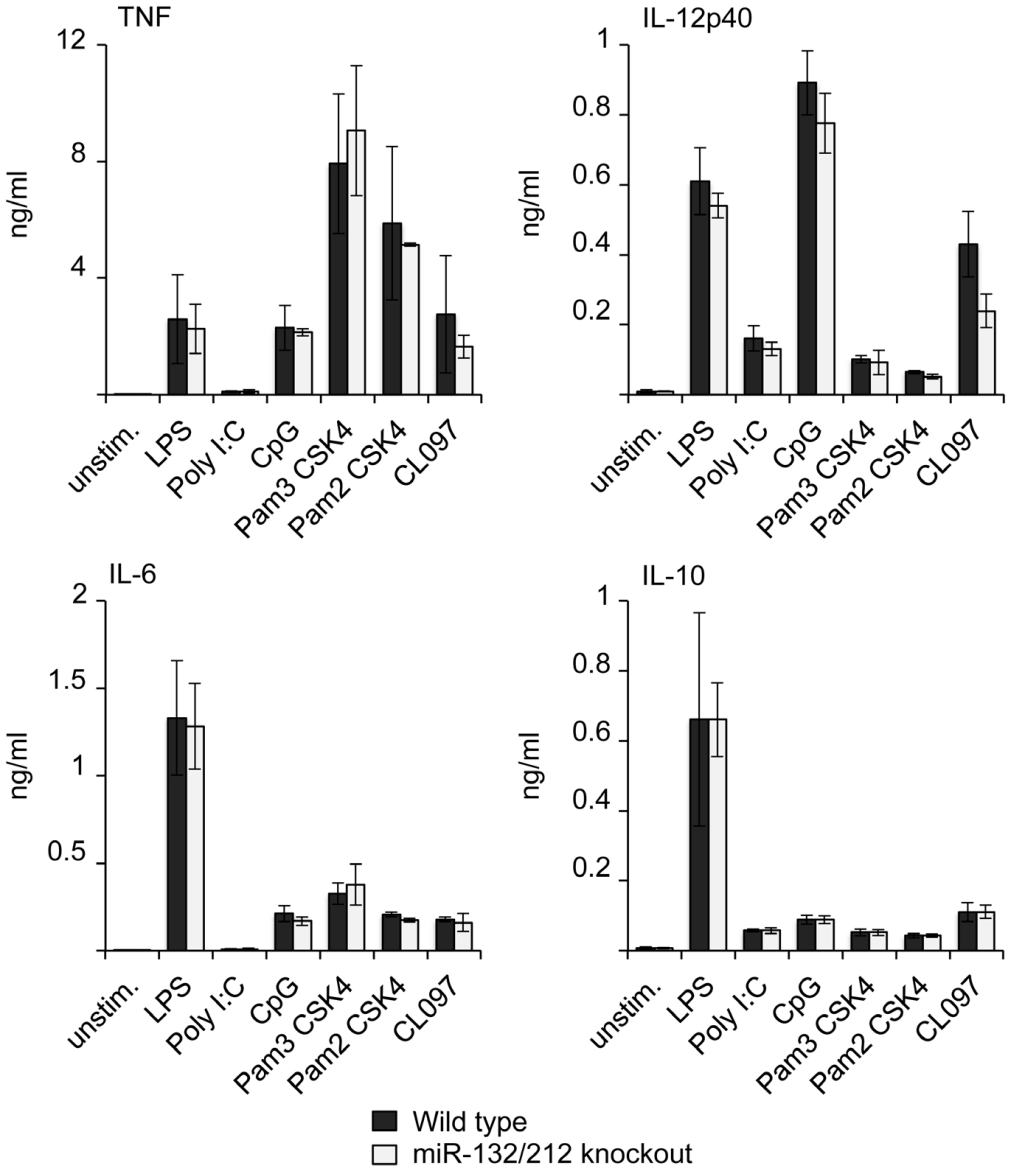
miR-132 and miR-212 are not required for cytokine induction in BMDMs. BMDMs were isolated from wild-type or miR-132/212 knockout mice. Cells were either left unstimulated or stimulated with 100 ng/ml LPS, 10 µg/ml poly I:C, 2 mM CpG, 100 ng/ml Pam3-CSK4, 100 ng/ml Pam3-CSK4 or 100 ng/ml CL097 for 6 h. Secreted levels of TNF, IL-12p40, IL-6 and IL-10 in the media were measured by a multiplex based assay as described in the methods. Error bars represent the standard deviation of independent cultures from 4 mice per genotype.

**Figure 5:**
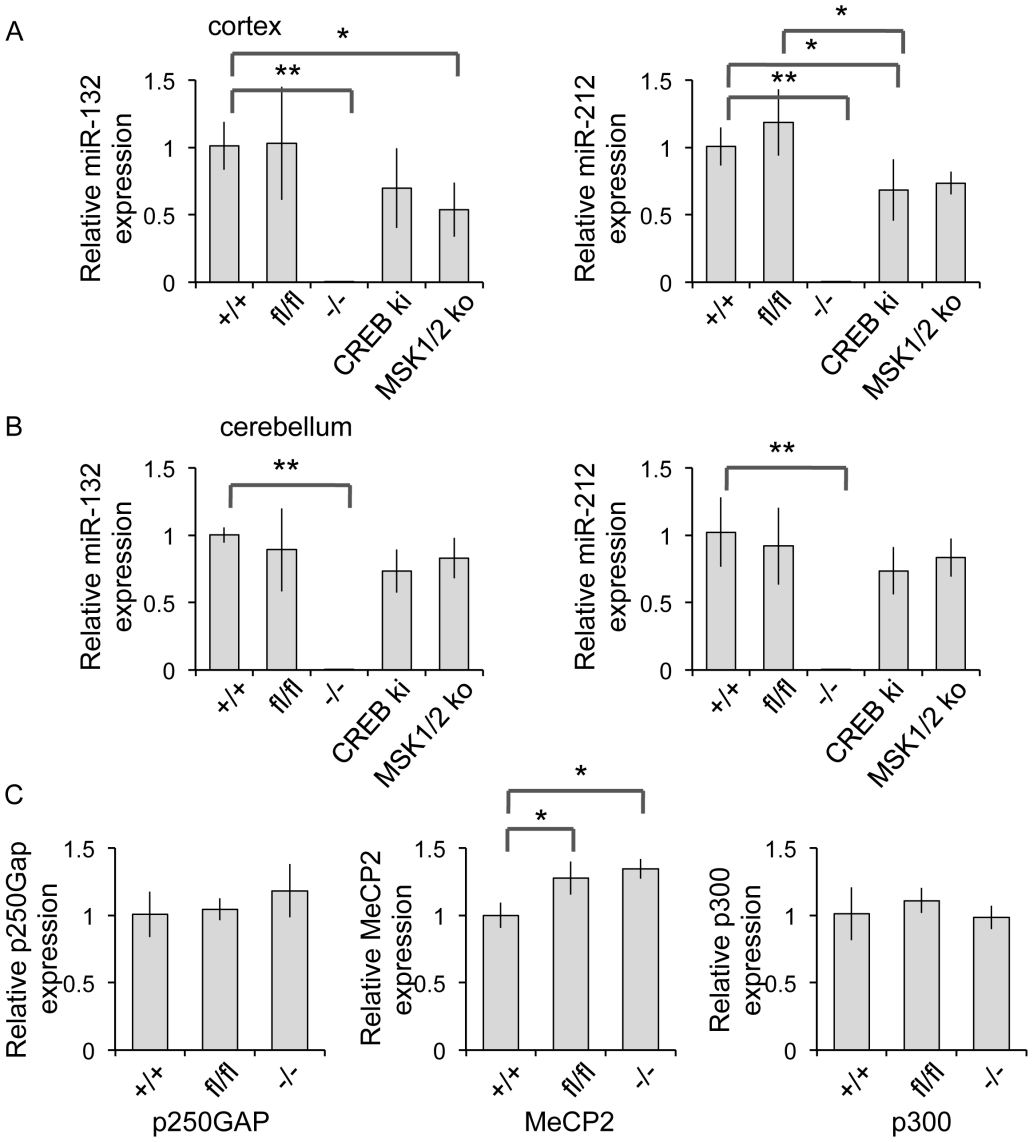
miR-132 and miR-212 do not regulate LPS-induced cytokine production *in vivo*. Wild-type or miR-132/212 knockout mice were given an intraperitoneal injection with either PBS or LPS (2 mg/kg). The levels of TNF, IL-10, IL-12p70, IL-12p40, IL-13, KC, MCP1, Mip-1a and Mip-1b were then determined at 1, 4 or 8 h after the injection using a multiplex cytokine assay. Error bars represent the standard deviation of 4 to 6 mice per condition.

To further examine the roles for miR-132 or miR-212 in innate immunity, bone marrow derived macrophages (BMDMs) were isolated from the knockout mice and stimulated with a panel of TLR agonists including LPS (TLR4), CpG (TLR9), Pam3-CSK4 (TLR1/2), Pam2-CSK4 (TLR2/3) and CL097 (TLR7). No differences were seen between the miR-132/212 knockout and wild-type macrophages for the production of TNF, IL-6, Il-12p40 and IL-10 (Fig. 4). LPS has been shown to induce miR-132 in the THP-1 human monocyte cell line [8]. While we have previously been able to replicate this finding we were unable to show induction of pri-miR-132/212 in BMDMs in response to LPS [3], [29]. Although this is consistent with the normal cytokine responses in the miR-132/212 knockout BMDMs, it is possible that these miRNAs may play a greater role in different macrophage subtypes or other immune cells. To explore this possibility, mice were injected with a sub-lethal dose of LPS and plasma cytokine measurements made at 1, 4 and 8 h after infection. Knockout of miR-132 and 212 did not affect the induction of TNF, Il-10, IL-13, IL-12, KC, MCP-1, Mip-1a or Mip-1b (Fig. 5).

### miR-132/212 Knockout Affects Synaptic Strength and Plasticity

In addition to its proposed roles in immunity miR-132 has been suggested to play roles in neuronal development and synaptic function [5], [16], [17], [30], [31]. For instance overexpression of miR-132 mimetics or inhibitors have been shown to have major effects on neuronal morphology in culture [4].

**Figure 6:**
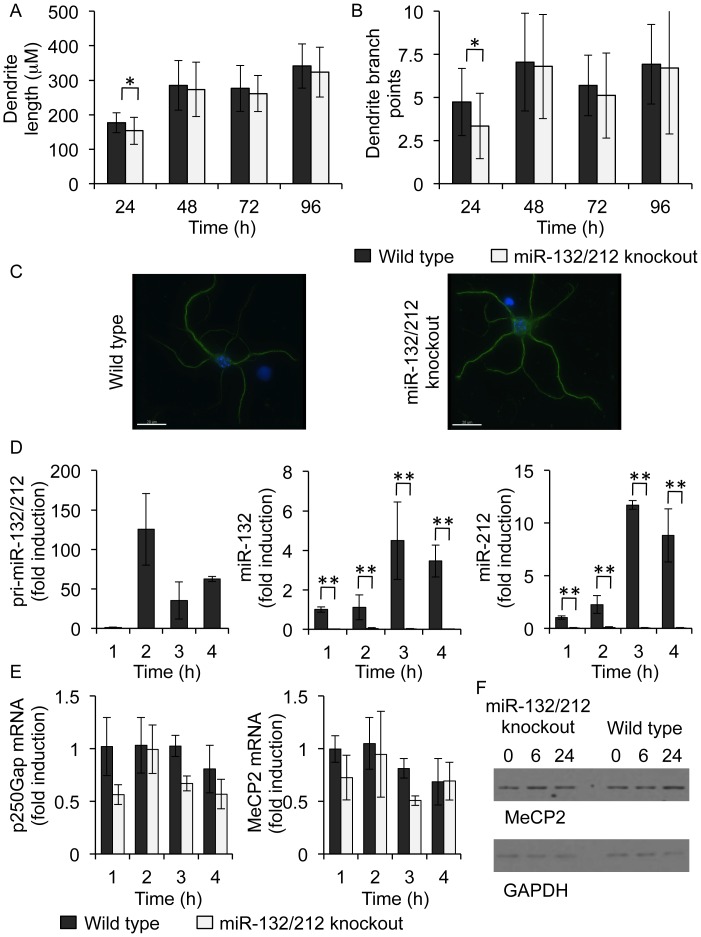
miR-132/212 knockout cortical cultures exhibit normal morphology. Cultures of cortical neurons were established on coverslips from P0 pups from either wild-type or miR-132/212 knockouts. After 24, 48, 72 or 96 h in culture, neurons were fixed with PFA and stained with MAP2 primary mouse monoclonal then fluorescein-labelled secondary anti-mouse antibody. Morphology was examined using confocal fluorescence microscopy and the images were analysed using the FilamentTracer Module of the Imaris software (Bitplane, Switzerland). Dendrite length (A) and branching (B) were quantified and representative images are shown with MAP2 staining in green and DAPI in blue (C). A p value (students t-test) between wild-type and knockout of <0.05 is indicated by * or <0.01 by **. Alternatively cultures were stimulated with 20 µM NMDA for the indicated times and total RNA isolated. The levels of pri-miR-132/212, miR-132, miR-212 (D), p250-Gap and MeCP2 (E) were determined. Error bars represent the standard deviation of 4 cultures per genotype. Similar experiments were also performed but the level of MeCP2 was determined by immunoblotting (E).

Analysis of cortical neuronal cultures demonstrated that knockout of miR-132 and miR-212 did not result in significant differences in neuronal morphology compared to wild-type control cultures between 2 and 4 days *in vitro*. However, there was a small decrease in dendrite length and branching 24 h after plating (Fig. 6A–C). Stimulation of the cultured neurons with the glutamate receptor agonist NMDA was able to increase miR-132 and miR-212 levels in wild-type cells (Fig. 6D). However, as expected, mature miR-132 and miR-212 could not be detected in the knockout cells (Fig. 6D). As judged by qPCR, miR-132/212 knockout did not significantly affect the levels of the potential miR-132 targets p250Gap or MeCP2 (Fig. 6E). Similar to the mRNA results, western blotting did not detect significant differences in the levels of MeCP2 protein in miR-132/212 knockout samples relative to wild-type cells (Fig. 6F). As we were unable to identify a good antibody to p250-Gap we were unable to determine the effect of the miR-132/212 knockout on the levels of p250-Gap protein.

Gross morphology of the brain by visual inspection was unaffected by miR-132/212 knockout (data not shown). In addition, analysis of spine density in mature CA1 hippocampal neurons did not reveal any differences between the control (fl/fl) (12.6±1.4 spines/10 µm) and miR-132/212 knockout (12.6±1.1 spines/10 µm; data from 2180 spines measured from 36 dendrites across 4 KO and 3 wild-type mice; data not shown).

**Figure 7:**
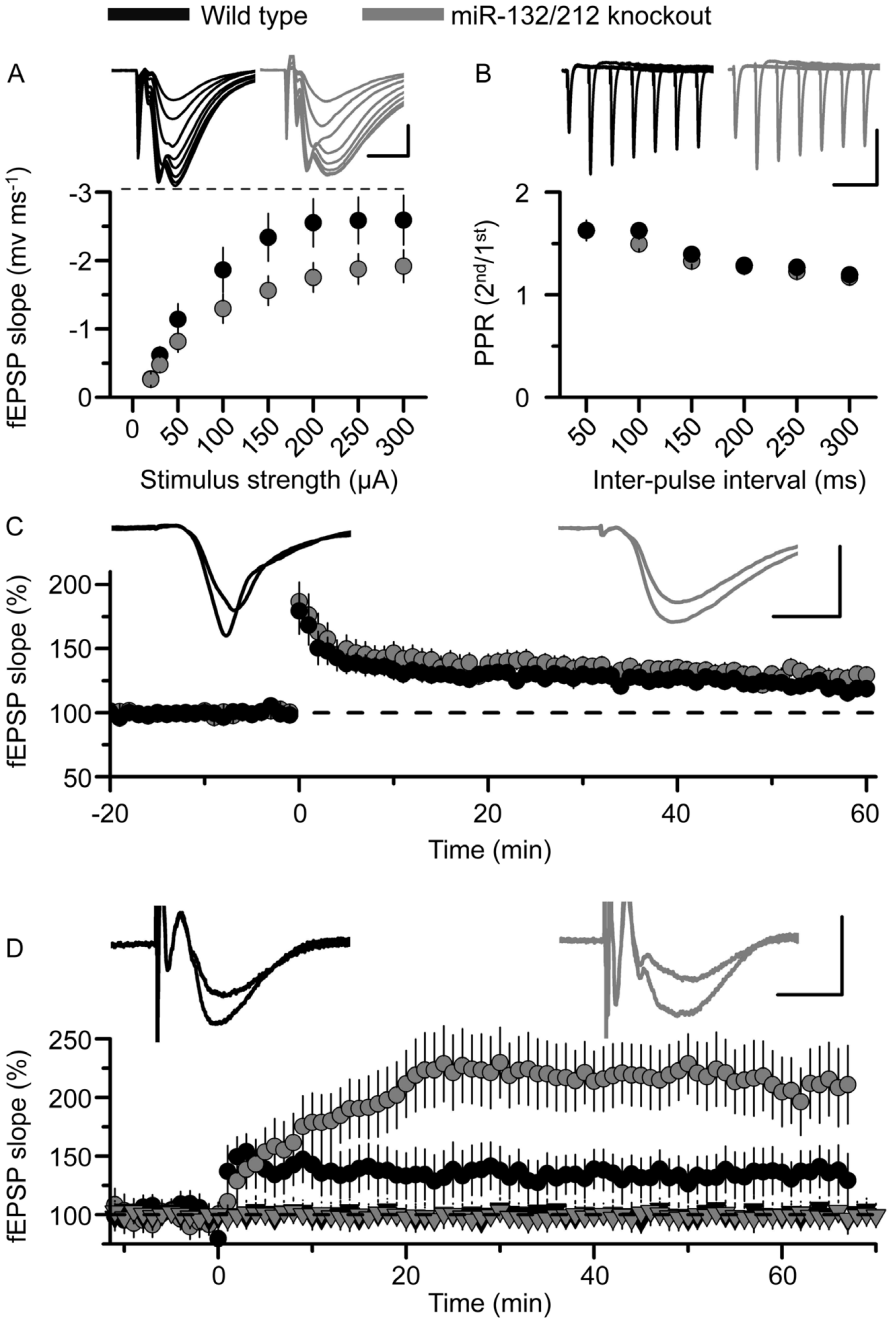
Deletion of miR212/132 influences hippocampal synaptic transmission and plasticity. A) Plot of fEPSP slope vs stimulus strength (mean ± SEM) showing that basal synaptic transmission is reduced by ∼30% in miR 212/132 KO slices (grey circles) compared to control (fl/fl) slices (black circles). Inset are representative fEPSPs at each of the stimulus strengths for fl/fl (left, black traces) and KO (right, grey traces) slices. Scale bar measures 5 ms and 1 mV. B) Paired-pulse facilitation (mean ± SEM) is no different between control (black circles) and miR KO slices (grey circles). Inset are representative fEPSPs at each of the inter-pulse intervals for control (left, black traces) and KO (right, grey traces) slices. Scale bar measures 100 ms and 1 mV. A and B represent data from 17 slices from 8 KO, and 11 slices from 6 control mice. C) LTP induced by tetanic stimulation (100 Hz for 1s) was not appreciably different between fl/fl (black circles) and KO mice (grey circles). Inset are fEPSPs taken before (smaller of the traces) and 60 mins after the induction of LTP (larger of the two traces) in fl/fl (left, black traces) and KO (right, grey traces) slices. Data from 11 slices from 8 KO mice and 6 slices from 4 control mice. Mean ± SEM. Scale bar measures 5 ms and 1 mV. D) In contrast, theta-burst stimulation (5 episodes at 10 s intervals of theta-burst stimulation (5 pulses at 100 Hz, repeated 10 times with 200 ms interval)) revealed greater LTP in KO slices (grey circles) compared to fl/fl (black circles) slices (p<0.05 at 55–65^th^ minute; unpaired t-test). Inset are fEPSPs taken before (smaller of the traces) and 60 mins after the induction of LTP (larger of the two traces) in fl/fl (left, black traces) and KO (right, grey traces) slices. The black and grey triangles hovering at 100% refer to the slope of fEPSPs from a simultaneously recorded control pathway for fl/fl and KO slices, respectively. Data from 13 slices from 5 KO mice and 6 slices from 2 fl/fl mice. Scale bar measures 5 ms and 1 mV. Data is presented as mean ± SD.

Next, we examined the effect of miR-132/212 on synaptic function. In these experiments to control for the insertion of the loxP site, homozygous miR-132/212 floxed mice were used as controls. These mice still express normal levels of miR-132 and miR-212 (Fig. 2). In acute hippocampal slices a deficit in synaptic transmission between CA3 and CA1 neurons was observed, with the knockout showing a consistently smaller fEPSP by ∼30% over a stimulation range of 50–300 µA (Fig. 7A), whilst paired-pulse facilitation, an indicator of the probability of vesicle release from the pre-synaptic terminal, was no different between miR-132/212 knockout mice and fl/fl mice (n = 17 slices from 8 KO and 11 slices from 6 fl/fl mice; Fig. 7B).

Long term potentiation (LTP) has been suggested to represent the cellular and molecular processes that underlie the formation of memory [32] LTP can be induced in a variety of regions in the brain, including in the CA3/CA1 pathway in the hippocampus. In hippocampal slices, LTP in the CA3/CA1 pathway induced by tetanic stimuli (100 Hz/1s) was unaffected by miR-132/212 knockout (n = 11 slices from 8 KO mice and 6 slices from 4 fl/fl mice; Fig. 7C). In contrast, LTP induced by theta burst stimulation was enhanced in the KOs relative to slices prepared from fl/fl mice (Fig. 7D).

**Figure 8:**
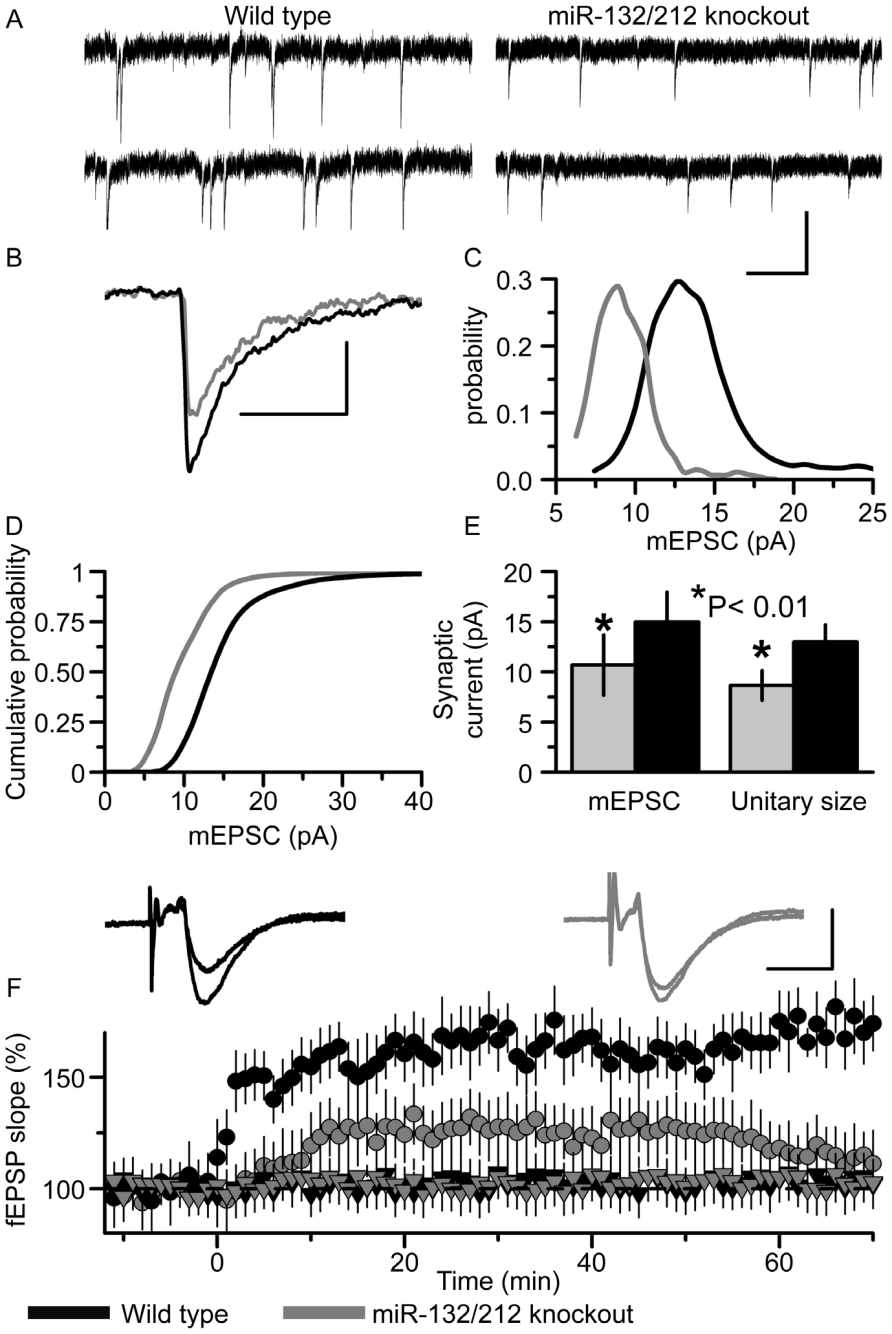
Deletion of miR132/212 influences synaptic transmission and plasticity in the neocortex. A) Representative examples of miniature spontaneous currents recorded in pyramidal neurons of the somatosensory cortex of a control (fl/fl mice, left panels) and miR-132/212 KO slice (right panels). Whole-cell recording were made at membrane potential of −80 mV in the presence of TTX (1 µM) and picrotoxin (100 µM). Scale bar measures 1 s and 10 pA. B) The superimposition of the average waveform (25 each) of mEPSCs recorded in the fl/fl neuron (black line) and miR-132/212 KO neuron (grey line) shown in A. Scale bar measures 20 ms and 5 pA. C) Superimposition of corresponding amplitude distributions of mEPSCs recorded in the fl/fl neuron (black) and miR-132/212 KO (grey) neuron. The leftward shift in the peak of amplitude distribution of mEPSCs in the KO neuron clearly indicates at decrease in the unitary size of the mEPSCs. D) Cumulative distributions of mEPSCs amplitudes pooled for 7 fl/fl (black line) and 8 miR-132/212 KO neurons (grey line) showing a consistent leftward shift towards reduced mEPSC amplitude in miR-132/212 KO neurons. E) Pooled data of average mEPSC and quantal, unitary size (mean ± SD for 7 fl/fl and 8 KO neurons). The difference in the average amplitude and quantal size between fl/fl and miR KO neurons was statistically significant with p<0.01 (ANOVA). F) Time course of changes in the slope of fEPSPs in layer 2/3 of somatosensory cortex after theta-burst stimulation (5 episodes at 10 s intervals of theta-burst stimulation (5 pulses at 100 Hz, repeated 10 times with 200 ms interval)). Each point represents the mean ± SD for 11 cortical slices of fl/fl mice (black circles) and 14 cortical slices of miR-132/212 KO mice (grey circles). The insets show the average fEPSP (20 each) waveforms recorded before (smaller of the two traces) and 60 min after theta-burst stimulation (larger of the two traces) in cortical slices of fl/fl (black lines) and miR-132/212 KO (grey lines) mice. The black and grey triangles hovering at 100% refer to the slope of fEPSPs from a simultaneously-recorded control pathway for fl/fl and KO slices, respectively. Long-term potentiation of excitatory synaptic transmission is impaired in the neocortex of miR-132/212 KO mice. Scale bar measures 5 ms and 1 mV.

In keeping with the synaptic transmission deficit observed in the hippocampus, there was also a similar significant deficit in synaptic transmission in the neocortex. The average amplitude and quantal size of glutamatergic mEPSCs recorded in pyramidal neurons of somatosensory cortex of miR-132/212 knockout mice was almost 40% lower than mEPSCs recorded from neurons of fl/fl mice (Fig. 8A, B). Analysis of the mEPSC amplitude distributions (see Methods) revealed a significant deficit in postsynaptic efficacy, evaluated by the quantal size of synaptic currents (Fig. 8C), whilst the cumulative distribution of mEPSC amplitudes in knockout mice was shifted to the left indicating a greater proportion of smaller synaptic events (Fig. 8D). The average mEPSC amplitude in neocortical neurons of miR-132/212 KO mice measured 10.5±3.1 pA (n = 8) as compared to average mEPSC amplitude of 14.9±2.9 pA (n = 7; p<0.01) in fl/fl mice (Fig. 8E). The average quantal size of mEPSCs in miR-132/212 KO mice was 8.4±1.4 pA (n = 8) whereas the same parameter for fl/fl neurons was 12.9±1.7 pA (n = 7; p<0.01; Fig. 8E). Furthermore, KO mice had reduced mEPSC frequency compared to fl/fl mice (0.45±0.17 Hz vs 0.76±0.29 Hz, mean ± SD, n = 7–9, p<0.02; one way ANOVA; data not shown). These data strongly suggest that the observed difference in the amplitude of the synaptic current is related to a decrease in the number of postsynaptic glutamate AMPA receptors. In contrast to hippocampal LTP, LTP induced in the neocortex by theta-burst stimulation was found to be significantly decreased in the miR-132/212 knockout (Fig. 8F).

Together these results point to a defect in synaptic function in both the hippocampus and neocortex of these mice, and potentially of a specific LTP deficit in neocortex.

## Discussion

We describe here the generation and initial characterisation of a miR-132/212 knockout mouse. Another miR-132/212 knockout has also recently been reported which used a similar strategy by replacing the miRNA sequence with a LacZ reporter gene. In that study homozygous matings for the knockout resulted in a high rate of postnatal death that was attributed to a defect in mammary gland development in the knockout females [10]. In contrast, breeding performance of the knockout females in our study was similar to that of heterozygous or wild-type females. While we did not specifically address mammary gland development in our mice, any phenotype present may be less severe than the one reported by Anand *et al* (2010). The reason for this could relate to genetic background or presence of the LacZ reporter in one of the mouse models. While our mice were generated using C57Bl/6 ES cells, Anand *et al* (2010) targeted in 129SvJ cells and then either maintained the mice on 129SvJ or backcrossed to C57Bl/6. In relation to this there are several genes, such as Nos2 and Nme1, close to miR-132 on chr11 that have been implicated in mammary gland development [33], [34]. It is possible that this could explain differences in severity between the knockout generated with C5/Bl6 ES cells and the lines transferred onto a C57Bl/6 background by backcrossing.

Previous studies have suggested roles for miR-132 in the CNS and immune system. In the immune system, roles for miR-132 have been proposed in the regulation of NFκB, the response to viral infection and in the regulation of immune responses [11], [13], [20], [35]. Despite this, initial analysis of the miR-132/212 knockout has not yet revealed any major defects in the innate immune system. Responses to viral infection or TLR agonists in primary cells from the mice were normal, as was the response to an LPS challenge *in vivo*. Lagos *et al*. (2010) reported that miR-132 was induced in response to infection with Kaposi’s sarcoma-associated herpes virus (KSHV) in human lymphatic endothelial cells. In their study it was found that miR-132 inhibited the anti-viral response to KSHV at least in part by targeting the transcriptional co-activator p300. As miR-132 is induced by CREB and p300 is a co-activator for CREB, this suggests that induction of miR-132 could set up a negative feedback loop to limit CREB-dependent transcription [13]. Following infection of fibroblasts with Sendai virus, the RNA levels of the CREB-dependent genes nur77 and pri-miR-132/212 were repressed suggesting that Sendai virus may inhibit CREB function. Significantly however, this was independent of miR-132 and miR-212 indicating that in this system it did not require modulation of p300 levels by miR-132. Additionally miR-132 did not affect the levels of IFNβ mRNA induced in response to Sendai virus infection. These differences could be due to several reasons; Sendai virus is an RNA virus while KSHV is a DNA virus and it is possible that miR-132 is only involved in the response to a subset of viruses or cell types. It is also possible that this is due to a species difference between the human and mouse immune response.

Recently it has been demonstrated that knockout of miR-132 can affect the dendritic growth and arborization of newborn neurons in the hippocampus [9] while similar results have been observed following retroviral expression of a miR-132 inhibitory sponge complex [36]. In line with this, transfection of miR-132 inhibitory oligos or miR-132 mimetics affects these processes in cultured neurons [4], and miR-132 over-expression increased the number of mushroom and stubby spines, but decreased overall spine number [17]. In contrast, in cultured cortical neurons we did not observe major differences between the morphology of wild-type and miR-132/212 knockout neurons, while spine density in the mature CA1 hippocampal neurons was similar. Together this would suggest that while miR-132 may play a role in newly developing neurons in the CNS, over time neurons can compensate for the loss of miR-132 in terms of dendrite outgrowth and spine formation.

Electrophysiological experiments however do indicate that miR-132 or miR-212 play a role in synaptic function. Overexpression of miR-132 has been shown to both increase paired-pulse facilitation but not mEPSC amplitude, suggesting a presynaptic action on the probability of neurotransmitter release [31], and increase both mEPSC amplitude and frequency [17]. Inhibition of miR-132 using retroviral constructs impaired synaptic transmission in hippocampal dentate gyrus granule cells, with a net effect of a reduction in the amplitude of evoked EPSCs, which likely reflects a reduction in the number of synapses since neither mEPSC amplitude nor the probability of transmitter release was affected, whereas the frequency of mEPSCs was greatly reduced. [36]. In the miR-132/212 knockout reported here, normal paired-pulse facilitation, and hence probability of transmitter release was observed in the hippocampus, but the amplitude of both evoked and spontaneous synaptic transmission was decreased in the hippocampus and neocortex. Quantal analysis of mEPSCs in the neocortex revealed a reduction in the amplitude of mEPSCs, together with a reduction in mEPSCs frequency. These observations are consistent with previous miR-132 over-expression studies [17] and suggest that miR-132/212 regulates cell surface expression of post-synaptic AMPA receptors at these synapses.

LTP is often used as a molecular model to explain the formation of memories, and the defects in the encoding of memory in knockout mice normally correlates with defects in LTP [32], [37]. LTP can be induced in different regions and neuronal types in the brain, and it is likely that there are differences in the molecular mechanism underlying different forms of LTP [38]. The expression of both miR-132 and miR-212 has been shown to be increased after the induction of LTP by high frequency stimulation in the dentate gyrus. Interestingly, these studies also showed that while the miR-132 levels were increased, this did not affect the protein levels of the putative miR-132 targets MeCP2 or p250GAP [6], a finding that is consistent with our observation that miR-132/212 knockout did not affect the expression levels of MeCP2 and p250GAP in cultured neurons (Fig. 6). In the present study we found that LTP in area CA1 of hippocampal slices induced by conventional tetanic stimulation (100 Hz/1sec) was no different between control and miR-132/212 knockout mice, whereas LTP induced by theta-burst stimulation was enhanced in miR-132/212 knockouts. In contrast, theta-burst LTP in the neocortex was reduced in the miR-132/212 knockouts relative to controls. Whilst the reason for this difference is unclear at present, it may involve differential signaling or GluA subunit expression and trafficking requirements for LTP at the two synapses. Considerable evidence already exists for such a differential in LTP induction and expression mechanisms, with the neocortex being more sensitive to pharmacological or genetic manipulations affecting LTP [38], reflecting the observations made in the present study.

Nonetheless, the effect of the miR-132/212 on cortical LTP is perhaps significant, given the recent findings that miR-132 is involved in plasticity in the visual cortex [23], [24]. It would therefore be of interest to examine the effects of miR-132 knockout in models of monocular deprivation.

In summary, miR-132 and miR-212 are two related miRNAs that can be induced by a variety of signals and in various cell types and have proposed functions in both the CNS and immunity. Through the generation of a miR-132/miR-212 double knockout, we have shown that these miRNAs are not essential for development or fertility. Initial experiments on the innate immune system of these mice have not revealed any significant phenotype. Analysis of neuronal systems suggests while they may have some developmental roles they are not critical for the formation of the CNS. miR-132 or miR-212 do however have roles in regulating synaptic transmission and synaptic plasticity, and it would therefore be of interest to examine the effect of miR-132/212 knockout in behavioural models.

## Methods

### Mice

miR-132/212 knockout mice were generated using the strategy described in Fig. 1A by TaconicArtemis. Briefly, the targeting vector was designed to introduce LoxP sites either side of the region encoding miR-132 and miR-212. A neomycin resistance cassette was included for positive selection and a TK cassette for negative selection. The sequence of this vector is provided as supplemental data. Targeting was carried out in ES cells derived from C57Bl/6N mice using standard protocols. Correctly targeted clones were identified by Southern blotting of Kpn I digested genomic DNA using a probe external to the targeting vector. Targeted ES cells were injected into blastocysts to generate chimeric mice. Germline transmitting chimeric mice were crossed to Flpe transgenic mice (also on a C57Bl/6 background) to delete the neomycin cassette, resulting in mice with a conditional allele for miR-132 miR-212. The deletion of miR-132 and miR-212 was achieved by crossing these mice to transgenic mice expressing Cre recombinase under a constitutive promoter (Taconis Artemis), following deletion mice were crossed away from the Cre transgene before experimental mice were generated. Routine genotyping of the mice was carried out by PCR using ear biopsy tissue. Reactions contained 3 primers (p1 ACGACAGACAGACGCACACCTC, p2 CTAGTCGAGGTATCGCTGCCTAAG, p3 TGAGGGAAGACTGCTGGCTGATAC) which gave rise to bands of 373 bp for the wild-type allele, 420 for the floxed allele and 550 for the deleted allele.

Wild-type C57Bl/6 mice were obtained from Charles River Laboratories or bred in house. Knockout mice for MSK1 and MSK2 and CREB Ser133Ala knockin mice have been described previously [27], [39], [40]. All mice were maintained in IVCs under specific pathogen free conditions. Mice were maintained in accordance with UK and EU regulations, and work was covered by an appropriate Home Office license (60/3923) which was subject to review by the University of Dundee Ethical Review Committee.

### Cell Culture

Bone marrow-derived macrophages were cultured as described [41]. Briefly bone marrow was flushed from the femurs of mice and cultured on bacterial grade tissue culture plates for 7 days in DMEM supplemented with 10% (vol/vol) FBS, penicillin (100 units/ml), streptomycin (100 µg/ml), amphothericin B (0.25 µg/ml), L-glutamine (2 mM) and mouse recombinant macrophage colony-stimulating factor (5 ng/ml; R&D Systems). Cells were then replated on tissue culture plastic and stimulated within 24 h of replating. BMDMs were stimulated with 2 mM CpG, 100 ng/ml Pam3-CSK4, 100 ng/ml Pam2-CSK4, 10 µg/ml poly I:C, 100 ng/ml CL097 or 100 ng/ml LPS as indicated. Embryonic fibroblasts were isolated as described [42] and cultured in DMEM supplemented with 10% FBS supplemented with 10% FBS, penicillin (100 units/ml), streptomycin (100 µg/ml), and L-glutamine (2 mM). Where indicated cells were stimulated with 400 ng/ml PMA or infected with 100 U/ml Sendai virus (Charles River Laboratories). Primary cortical neurons were isolated from P0 pups as described and grown in Neurobasal A (Invitrogen) supplemented with 2% B27, 1% FBS, L-glutamine (0.4 mM), penicillin (100 U/ml) and streptomycin (0.1 µg/ml) and plated on poly-D-lysine-coated plates (100 µg/ml; Sigma) [28].

### Quantitative PCR

Total RNA was isolated using microRNeasy mini kits (Qiagen) in line with the manufacturer’s protocol. For qPCR of mRNA or intron sequences, total RNA was reverse-transcribed with iScript (Bio-Rad Laboratories) in line with the manufacturer’s protocols. qPCR was carried out using SYBR green detection methods. Fold induction was calculated relative to the unstimulated control (wild-type) sample, using 18S or GAPDH levels to correct for loading. Primer sequences are given in Table 1. qPCR for mature miRNA was carried out using TaqMan MicroRNA assays from Applied Biosystems, according to the manufacturer’s protocols. miR-16 levels were used to correct for total RNA levels.

**Table 1:** Primer sequences used for qPCR.

gene	sense	antisense
pri-miR-132/212	CGGTGACTCAGCCTAGATGG	GGACGGGACAGGGAAGGG
p250-Gap	AGAGGTATGGCATTGTGGATGG	GTAGGTGAGCAGAGGGTTTGG
Nur77	CCTGTTGCTAGAGTCTGCCTTC	CAATCCAATCACCAAAGCCACG
IFNβ	GGAAAAGCAAGAGGAAAGATTGAC	CCACCATCCAGGCGTAGC
18s	GTAACCCGTTGAACCCCATT	CCATCCAATCGGTAGTAGCG
GAPDH	ACAGTTCTTCATGTGGTGACCC	TGCACCACCAACTGCTTAG
MeCP2	Mm_Mecp2_va.1_SG Quantitect primer assay (200) – QT01659448 (Qiagen)	
p300	Mouse EP300 Solaris qPCR Gene expression assay – AX-065607-00-0100 (Dharmacon)	

### Immunoblotting

Cortical neurons or BMDMs were lysed directly into SDS sample buffer (1% (w/v) SDS, 10% (v/v) glycerol, 50 mM Tris–HCl pH 7.5, 1 mM EGTA, 1 mM EDTA, 1 mM sodium orthovanadate, 50 mM sodium fluoride, 1 mM sodium pyrophosphate, 0.27 M sucrose, 1% (v/v) Triton X-100, 0.1% (v/v) 2-mercaptoethanol). Samples were run on 10% polyacrylamide gels, and immunoblotted using standard techniques. The antibody against MeCP2 was from Abcam.

### Cytokine Measurement

Cytokines were measure using a multiplex based system from Bio-Rad according to the manufactures protocol. Data was acquired on a Luminex 100 system.

### Electrophysiological Recordings in Hippocampal Slices

Hippocampal slices were prepared as previously described [43] Briefly, control (fl/fl) or miR-132/212 knockout mice were killed by cervical dislocation in accordance with the UK Animal (Scientific Procedures) Act, 1986 and local Ethical Review procedures. The brain was rapidly removed and immersed in ice-cold artificial cerebrospinal fluid (aCSF) containing 10 mM Mg^2+^. Parasagittal slices (400 µm) were cut on a Microm vibratome and incubated at room temperature in aCSF (in mM): NaCl, (124); KCl, (3); CaCl_2_, (2); NaHCO_3_, (26); NaH_2_PO_4_, (1.25); D-glucose, (10); MgSO_4_, (1); pH 7.4 with 95% O_2_/5% CO_2_ and was gassed with 95% O_2_/5% CO_2_. Field excitatory postsynaptic potentials (fEPSPS) were recorded in area CA1 at 33–34°C with an aCSF-filled glass microelectrode following stimulation (0.1 ms duration; 15 s intervals) of the afferent Schaffer collateral-commissural pathway at the level of stratum radiatum with a twisted bipolar electrode made from 50 µm Teflon-coated tungsten wire. fEPSPs were sampled at 10 kHz and filtered between 1 Hz and 3 kHz. fEPSP acquisition and analysis was under the control of LTP software courtesy of Dr Bill Anderson and Prof Graham Collingridge (University of Bristol) [44].

Input/output curves of basal synaptic transmission were constructed using stimulation currents from 20–300 µA and paired-pulse facilitation determined using two pulses delivered at between 50 and 300 ms interpulse interval. LTP was induced by tetanic stimulation (100 Hz/1s) or by theta-burst stimulation (5 episodes of theta-burst stimulation delivered with 10 s interval; each episode consisted of 5 pulses of 100 Hz stimulation, repeated 10 times with 200 ms interval ie a total 50 pulses per episode) of the Schaffer pathway. Slices were excluded if the fEPSP fell below the previous baseline by 60 min post-tetanus. Tetanus-induced LTP studies were conducted on eight miR-132/212 knockout mice (11 slices) and four fl/fl mice (6 slices), whilst theta-burst LTP experiments were conducted on five miR-132/212 knockout mice (13 slices) and 2 fl/fl mice (6 slices). For hippocampal LTP experiments, fEPSPs in fl/fl and KO slices were matched at ∼1 mV. Recordings of basal transmission and paired-pulse facilitation (slope 2^nd^ fEPSP/slope 1^st^ fEPSP) were made from eight miR-132/212 knockout mice (17 slices) and six fl/fl mice (11 slices).

Dendritic spines in area CA1 were visualised using an Alexa Fluor 488 fluorescently-labelled 10 kDa dextran (Invitrogen; D-22910), which was injected into the pyramidal cell layer of area CA1 and left to diffuse into the dendrites of stratum radiatum for 3 hrs in slices that had been returned to a the incubation chamber after injection (usually 3–4 injection sites per slice) using a protocol described by Delaney [45] and used previously [46]. After incubation, slices were fixed (4% PFA), cryopreserved in 30% sucrose and cryo-sectioned at 20 µm. Fluorescently labelled sections were visualised using confocal z-stacks, spines identified and the number counted per unit length of dendrite. Data was obtained from sections from four miR-132/212 knockout mice and three fl/fl mice.

### Electrophysiological Recordings in the Neocortical Slices

Brain slices were prepared as described above; the thickness of slices used in the electrophysiological recordings was 300 µm. Whole-cell voltage clamp recordings from pyramidal neurons of neocortical layer 2/3 were made with patch pipettes (4–5 MΩ) filled with intracellular solution consisting of 110 mM KCl, 10 mM NaCl, 10 mM HEPES, 5 mM MgATP, 0.2 mM EGTA, pH 7.35. The membrane potential was clamped at −80 mV unless stated otherwise. Currents were monitored using an AxoPatch200B patch-clamp amplifier (Axon Instruments, USA) filtered at 2 kHz and digitized at 4 kHz. Experiments were controlled by PCI-6229 data acquisition board (National Instruments, USA) and WinFluor software (Strathclyde Electrophysiology Software, UK); data were analyzed by custom lab-designed software. Liquid junction potentials were measured with the patch-clamp amplifier; all voltages reported were corrected accordingly. Series and input resistances were respectively 5–7 MΩ and 500–1100 MΩ; both series and input resistance varied by less than 20% in the cells accepted for analysis.

For activation of synaptic inputs, axons originating from layer IV-VI neurons were stimulated with a bipolar coaxial electrode (WPI, USA) placed in the layer V close to the layer IV border, approximately opposite the site of recording; stimulus duration was 300 µs. The fEPSPs were recorded in neocortical layer 2/3 with an aCSF-filled glass microelectrode following the stimulation of the cortical afferents at 0.1 Hz with stimulus strength adjusted to evoked response of 40–50% of maximal amplitude (usually 12–25 µA) to give fEPSPs across both genotypes of ∼0.7 mV. Long-term potentiation of neocortical fEPSPs was induced by 5 episodes of theta-burst stimulation delivered with 10 s interval; each episode consisted of 5 pulses of 100 Hz stimulation, repeated 10 times with 200 ms interval (total 50 pulses per episode). This protocol was the same as that used for theta-burst stimulation in hippocampal slices.

All data are presented as mean ± SD, the statistical significance of difference between age groups was tested by one-way ANOVA test, unless indicated otherwise. The spontaneous transmembrane currents recorded in the neocortical neurons were analysed off-line using methods described previously [47], [48]. Briefly, the inward transmembrane currents of amplitude greater than 2 SD of baseline noise were selected for the initial detection of spontaneous events. Thereafter every single spontaneous event was analyzed within a 140 ms time window and its amplitude and kinetics were determined by fitting a model curve with single exponential rise and decay phases. As a rule, mean square error of fit amounted to 5–20% of peak amplitude depending on the background noise. Whenever the error of fit exceeded 25%, spontaneous currents were discarded from further analysis. The amplitude distributions of spontaneous and evoked currents were analyzed with the aid of probability density functions and likelihood maximization techniques, as described previously [47], [48]; all histograms shown were calculated as probability density functions.

All electrophysiological experiments were conducted blind to the genotype, the code for which was cracked after analysis had been concluded.
